# MiR-199a-3p decreases esophageal cancer cell proliferation by targeting p21 activated kinase 4

**DOI:** 10.18632/oncotarget.25375

**Published:** 2018-06-19

**Authors:** Pornima Phatak, Whitney M. Burrows, Ingrid E. Chesnick, Mohan E. Tulapurkar, Jaladanki N. Rao, Douglas J. Turner, Anne W. Hamburger, Jian-Ying Wang, James M. Donahue

**Affiliations:** ^1^ Department of Surgery, University of Maryland School of Medicine, Baltimore, MD 21201, USA; ^2^ Baltimore Veterans Affairs Medical Center, Baltimore, MD 21201, USA; ^3^ Department of Pathology, University of Maryland School of Medicine, Baltimore, MD 21201, USA; ^4^ Division of Pulmonary and Critical Care, University of Maryland School of Medicine, Baltimore, MD 21201, USA; ^5^ Department of Surgery, University of Alabama at Birmingham School of Medicine, Birmingham, AL 35294, USA

**Keywords:** microRNA, miR-199a-3p, PAK4, esophageal cancer

## Abstract

Although microRNA (miR) 199a-3p functions as a tumor suppressor in multiple malignancies, its expression and role in esophageal cancer have not been studied. Based on our previous observation that miR-199a-3p is markedly downregulated in esophageal cancer cell lines relative to esophageal epithelial cells, we examined the function of miR-199a-3p in these cells. MiR-199a-3p is predicted to bind with high affinity to the mRNA of p21 activated kinase 4 (PAK4). This kinase has been shown to be overexpressed in several malignancies and to modulate proliferation and motility. The current study is designed to determine whether miR-199a-3p regulates the expression of PAK4 in esophageal cancer cells and to understand the functional consequences of this interaction. Herein, we demonstrate reduced expression of miR-199a-3p in human esophageal cancer specimens and cell lines compared to esophageal epithelial cells, with associated increased expression of PAK4. Forced expression of miR-199a-3p decreases expression of PAK4 in esophageal cancer cell lines. Mechanistic studies reveal that miR-199a-3p binds to the 3’UTR of PAK4 mRNA. This interaction results in reduced levels of PAK4 mRNA due to decreased mRNA stability. Downregulation of PAK4 leads to decreased cyclin D1 (CD1) transcription and protein expression, resulting in markedly impaired cellular proliferation. When PAK4 expression is rescued, both CD1 transcription and protein return to baseline levels. Our results show that miR-199a-3p functions as a tumor suppressor in esophageal cancer cells through repression of PAK4. These findings suggest that both miR-199a-3p and PAK4 may be novel therapeutic targets in the treatment of esophageal cancer.

## INTRODUCTION

Over the past 10 years, the incidence of esophageal cancer has increased by 16% in the United States, with approximately 17,000 cases estimated to be diagnosed in 2018 [1–2]. Unfortunately, in the same time period, overall survival has only improved from 15% to 18% [1–2]. Esophageal cancer is now the seventh leading cause of death in men in the United States [2]. Unlike other malignancies, such as lung, breast, and colon, no targeted therapies have yet been developed specifically for esophageal cancer. Obtaining a more detailed understanding of the molecular mechanisms involved in the development and progression of esophageal cancer will be necessary in order to identify novel therapeutic targets.

MicroRNAs (miRs) are well recognized as critical post-transcriptional regulators of gene expression in cancer cells [3]. Because of the specificity of the miR-target interaction, analyzing the roles of individual miRs differentially expressed in esophageal cancer cells can be used to identify molecular targets that directly regulate processes that are critically involved in esophageal cancer oncogenesis. In a previously published array analysis comparing global miR expression in a human esophageal epithelial cell line (hESO) to the human esophageal squamous cancer cell lines TE7 and TE10, we found that miR-199a-3p was one of the most downregulated miRs in the esophageal cancer cells, with a decrease in expression of greater than 3 log fold [4].

Although its function has not been defined in esophageal cancer cells, miR-199a-3p has been demonstrated to function as a tumor suppressor in multiple malignancies and to regulate several critical oncogenic targets. In endometrial cancer, miR-199a-3p has been shown to target mTOR [5]. In both ovarian cancer and renal cell cancer, miR-199a-3p was found to regulate c-Met expression [6, 7]. In osteosarcoma, miR-199a-3p was found to target the receptor-tyrosine kinase AXL, while aurora kinase A was shown to be regulated by miR-199a-3p in prostate cancer [8, 9].

Based on miR-target prediction programs, miR-199a-3p is predicted to bind the mRNA of p21 activated kinase 4 (PAK4) with high affinity. PAK4 is a member of Group 2 of the p21 activated kinase family. These serine/threonine kinases are down-stream effectors of CdC42 and Rac1 and play important roles in cell proliferation, survival, and motility. Overexpression of PAK4 has been demonstrated in multiple malignancies including breast, prostate, and pancreas, although its role in esophageal cancer has not been well studied [10–12]. We hypothesized that reduced expression of miR-199a-3p in esophageal cancer cells may lead to increased expression of PAK4. This study was designed to determine the expression levels of miR-199a-3p and PAK4 in esophageal cancer cells, in both human specimens and a panel of cell lines. In addition, we characterized the interaction between miR-199a-3p and PAK4 mRNA in esophageal cancer cells and elucidated the phenotypic effects of modulating expression of miR-199a-3p in these cells.

## RESULTS

### Expression of miR-199a-3p and PAK4 in esophageal cancer specimens and cell lines

**Figure 1:**
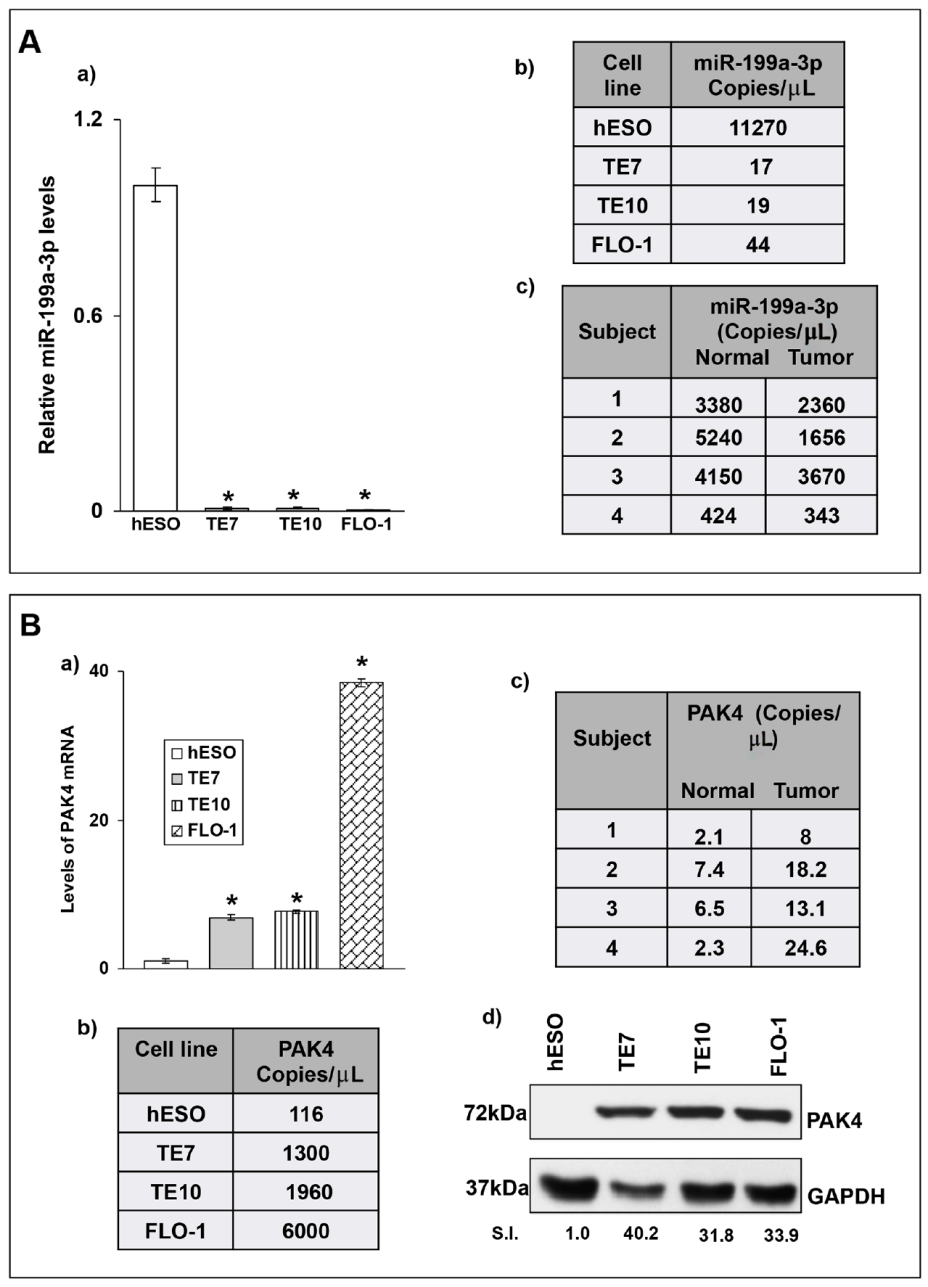
**(A)** Baseline miR-199a-3p levels in levels in human esophageal cell lines and human samples. (A, a) Endogenous relative miR-199a-3p expression levels in human esophageal cell lines as examined by q-PCR. MiR-199a-3p levels of human esophageal cancer cell lines (TE7, TE10 and FLO-1) were compared to miR-199a-3p levels of human esophageal epithelial cells (hESO). Total RNA was isolated from cells, followed by RT-q-PCR. Levels of miR-199a-3p were normalized with small nuclear RNA U6. A representative experiment of three independent experiments is shown. Error bars represents ± S.D. and statistical significance based on a two-tailed Student’s *t* test is indicated by ^*^ (p < 0.001). (A, b) Copy numbers of miR-199a-3p in esophageal cell lines shown in (A, a). (A, c) Copy numbers of miR-199a-3p in human esophageal cancer samples and matched benign esophageal epithelium. The copy numbers were measured using droplet digital PCR (dd PCR) technique and the concentration of miR was calculated in copies per microliter in each cell line and human specimen. **(B)** Levels of PAK4 are inversely correlated with miR-199a-3p levels. (B, a) Relative PAK4 mRNA levels in human esophageal cancer cell lines compared to hESO cells as examined by q-PCR. Levels of PAK4 mRNA for each cell line are normalized with GAPDH mRNA levels. Statistical significance is indicated by ^*^ (p < 0.001). (B, b) Copy numbers of PAK4 mRNA in esophageal cell lines shown in (B, a) measured by dd-PCR. (B, c) Copy numbers of PAK4 mRNA in human esophageal cancer samples and matched benign esophageal epithelium measured by dd-PCR. (B, d) Representative immunoblot forendogenous PAK4 protein levels in human esophageal cell lines shown in (B, a) GAPDH was used as a loading control. S.I. = Relative PAK4 protein mean signal intensity. Signal intensity of the target proteins is determined by densitometry and is normalized by signal intensity of GAPDH. Relative signal intensity (SI) for target protein is calculated compare to hESO.

In order to confirm our previous array data [4] showing marked downregulation of miR-199a-3p in the esophageal cancer cell lines TE7 and TE10 compared to esophageal epithelial cells, total RNA was harvested from these cells, as well as the human esophageal adenocarcinoma cell line, FLO1, and quantitative real-time PCR (q-PCR) analysis was performed. As seen in Figure 1A-1a, these data are in excellent agreement with the array analysis, with a marked reduction in miR-199a-3p levels in all three esophageal cancer cell lines. When copy number is assessed in these cell lines by droplet PCR (dd-PCR), miR-199a-3p is found to be downregulated in the cancer cell lines by approximately 3 log orders relative to hESO cells (Figure 1A-1b)

To investigate the clinical relevance of our findings, we measured miR-199a-3p levels in four human esophageal cancer specimens and matched benign esophageal epithelium. Importantly, none of these patients received chemotherapy or radiation therapy prior to surgery. Total RNA was extracted and subjected to dd-PCR analysis to determine copy number. As seen if Figure 1A-1c, mean copy numbers for miR-199a-3p are reduced in tumor tissue compared to matched benign esophageal epithelium in all four patients.

We next assessed differences in expression of PAK 4 in these specimens and cell lines. Levels of PAK4 mRNA are markedly elevated in all three cancer cell lines compared to hESO cells as measured by both q-PCR and dd-PCR (Figure 1B-1a, 1b). In the human specimens, there is a similar increase in PAK mRNA levels in the tumor samples compared to matched normal controls (Figure 1B-1c). Finally, PAK4 protein is markedly overexpressed in all three cancer cell lines compared to hESO cells (Figure 1B-1d). Based on these results, we chose to further evaluate the relationship between miR-199a-3p and PAK4 in esophageal cancer cells.

### Modulating miR-199a-3p levels leads to alterations in PAK4 expression and mRNA stability

**Figure 2:**
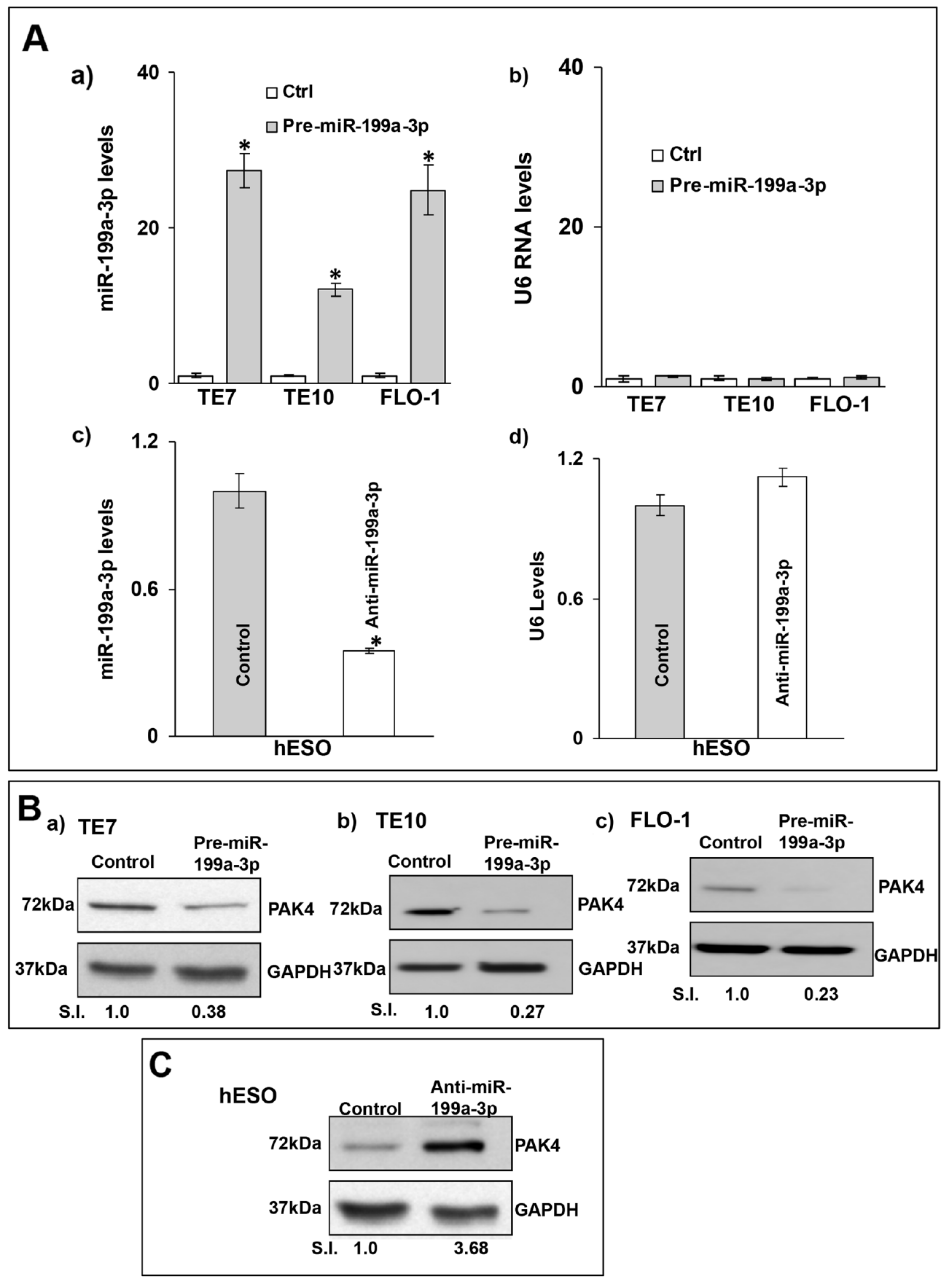
MiR-199a-3p reduces PAK4 expression in human esophageal cells **(A)** Cells were transfected with control miR or (A, a) with 50 nM pre-miR-199a-3p (TE7 TE10 and FLO-1) or (A, c) with 50 nM anti-miR-199a-3p (hESO). Forty-eight hours post-transfection, levels of miR-199a-3p (A, a and c) and U6 (A, b and d) RNA were measured by q-PCR. Values are mean ± SD from three independent sets of experiment in triplicate. Statistical significance is indicated by ^*^ (p < 0.002). **(B)** In similar experiments, whole cell lysates were subjected to Western blot analysis for PAK4 protein levels in (B, a) TE7, (B, b) TE10 and (B, c) FLO-1 cells, following control miR or 50 nM pre-miR-199a-3p overexpression. **(C)** Western blot analysis of PAK4 expression in hESO cells following transfection with control miR or 50 nM anti-miR-199a-3p. Representative immunoblots of three independent experiments in all the cell lines. Relative signal intensity was calculated as explained in Figure 1B-d.

Because basal levels of miR-199a-3p are low in all three esophageal cancer cell lines, transfection of pre-miR-199a-3p into these cells was performed in order to assess the effects on PAK4 expression. In reciprocal experiments, anti-miR-199a-3p was employed to reduce miR-199a-3p levels in hESO cells. Transfection efficiency of pre-miR-199a-3p was robust in the esophageal cancer cells. Similarly, transfection of anti-miR-199a-3p was very effective in reducing miR-199a-3p levels in hESO cells (Figure 2A-2a, 2c). Following successful transfection of pre-miR-199a-3p, protein levels of PAK4 were markedly decreased in all three cancer cell lines (Figure 2B). Conversely, PAK4 protein levels were increased compared to control-miR transfection in hESO cells following transfection of anti-miR-199a-3p (Figure 2C).

**Figure 3:**
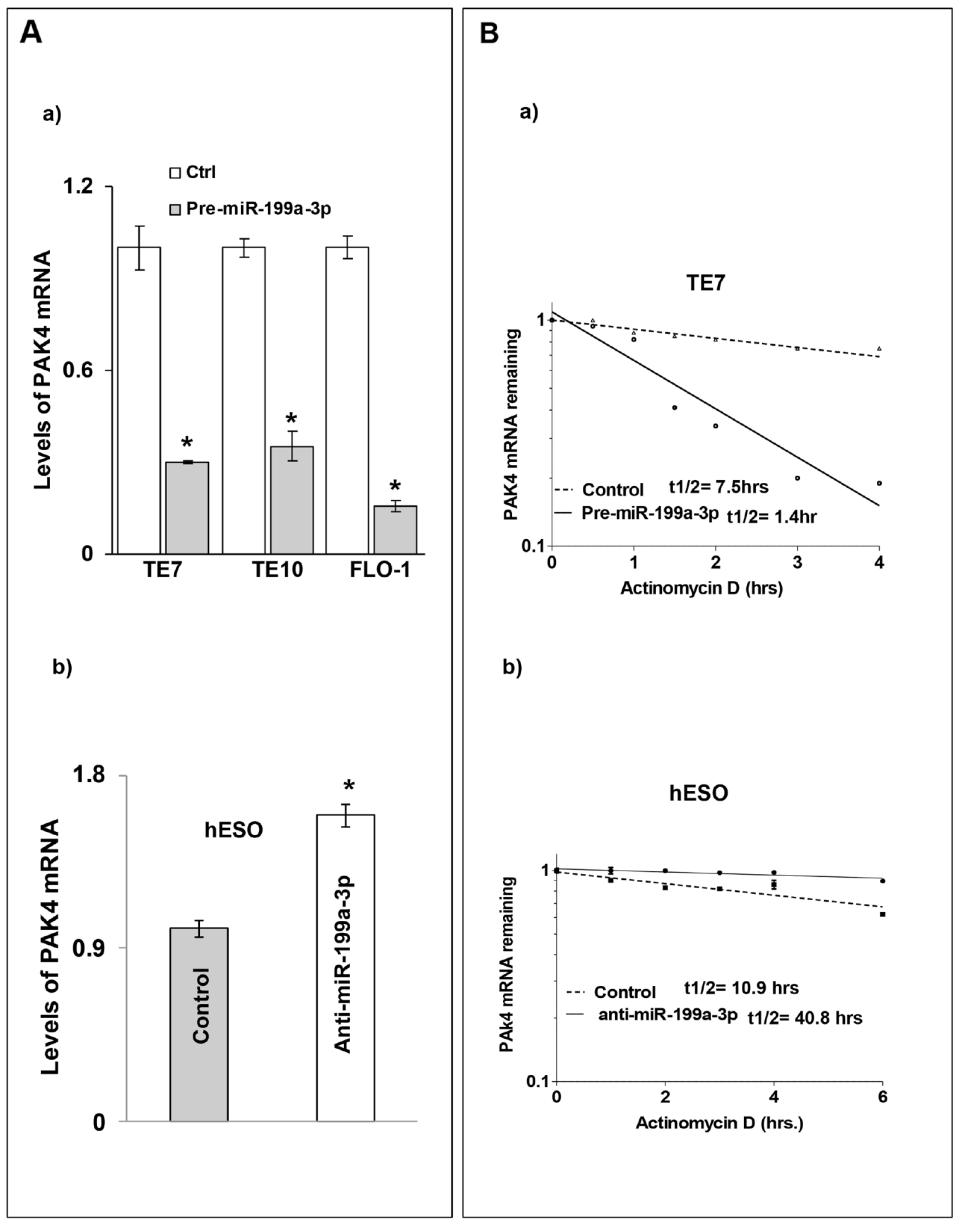
MiR-199a-3p reduces PAK4 mRNA levels and stability in human esophageal cells **(A)** Cells were transfected with control miR or (A, a) with 50 nM pre-miR-199a-3p (TE7, TE10 & FLO-1) or (A, b) with 50 nM anti-miR-199a-3p (hESO). Forty-eight hours post-transfection, levels of miR-199a-3p and U6 RNAwere measured by q-PCR. Values are mean ± SD from three independent sets of experiments performed in triplicate. Statistical significance is indicated by ^*^ (p < 0.001). **(B)** Stability of PAK4 mRNA. Following transfection of either control miR or 50 nM pre-miR-199a-3p in TE7 cells (B, a) or 50 nM anti-miR-199a-3p (hESO) (B, b), total RNA was isolated at indicated time points after administration of actinomycin D (4μM). The remaining levels of PAK4 mRNA were measured by q-PCR. PAK4 mRNA levels were normalized with GAPDH. The half-life was calculated from the first order equation t_1/2_ = ln2/k. Each point is the mean ± S.D. of three separate experiments.

To understand the mechanism by which miR-199a-3p affects PAK4 protein expression, levels of PAK4 mRNA were measured following overexpression of pre-miR-199a-3p in the cancer cells, as well as following transfection of anti-miR-199a-3p in hESO cells. As seen in Figure 3A-3a, transfection of pre-miR-199a-3p was associated with a significant decrease in PAK4 mRNA levels in all three cancer cell lines. As anticipated, reduction of miR-199a-3p expression in hESO cells led to increased PAK4 mRNA levels (Figure 3A-3b).

We next determined stability of PAK4 mRNA following modulation of miR-199a-3p expression. In these experiments, 48 hours following transfection, cells were exposed to 4 μM of Actinomycin D to inhibit further transcription. Total cellular RNA was harvested at specified time points and levels of PAK4 mRNA were measured by q-PCR. As seen in Figure 3B-3a, PAK4 mRNA stability is markedly decreased in TE7 cells following pre-miR-199a-3p transfection. Silencing miR-199a-3p in hESO cells resulted in an increase in PAK4 mRNA stability compared to transfection with control miR (Figure 3B-3b).

### MiR-199a-3p binds to PAK4 mRNA

**Figure 4:**
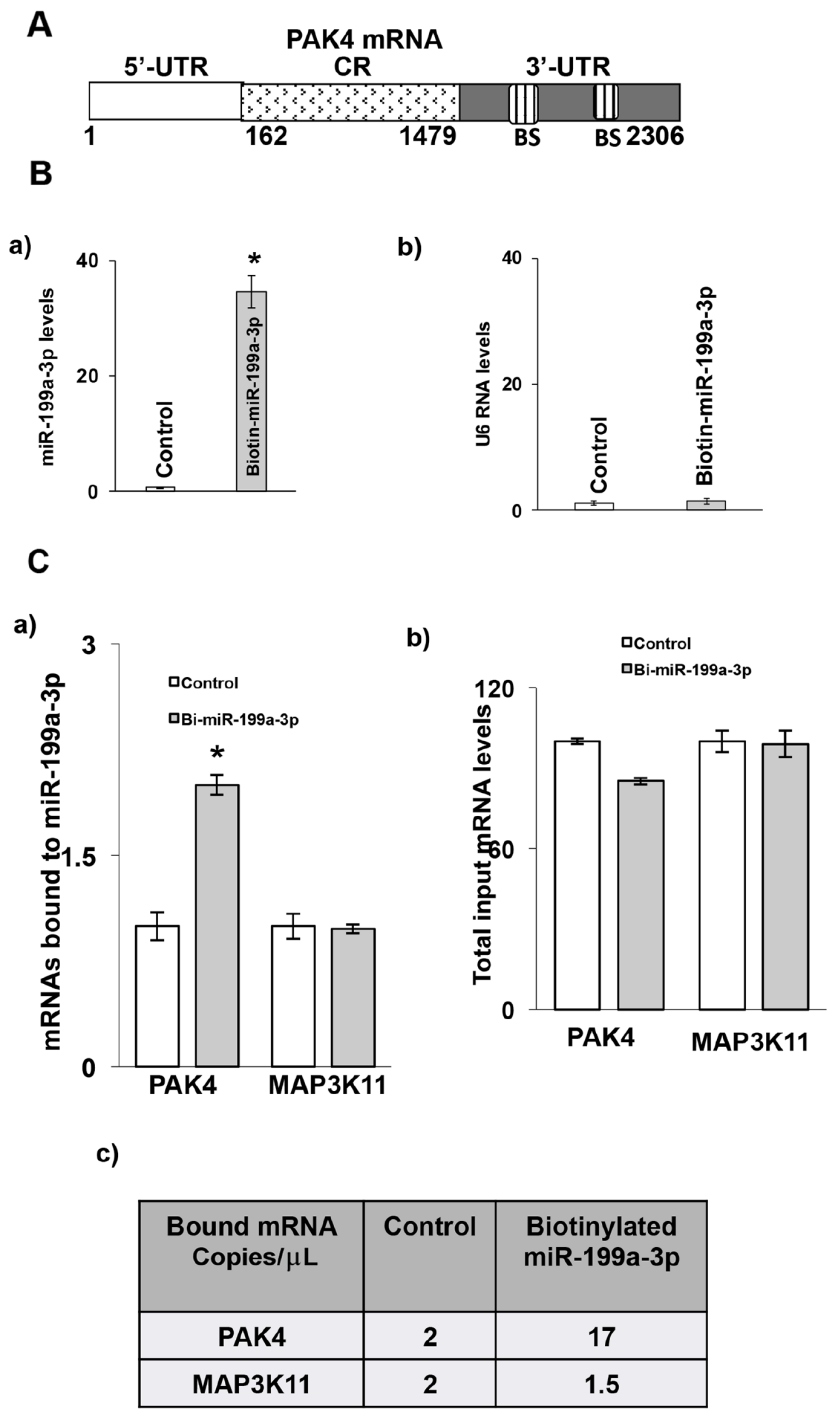

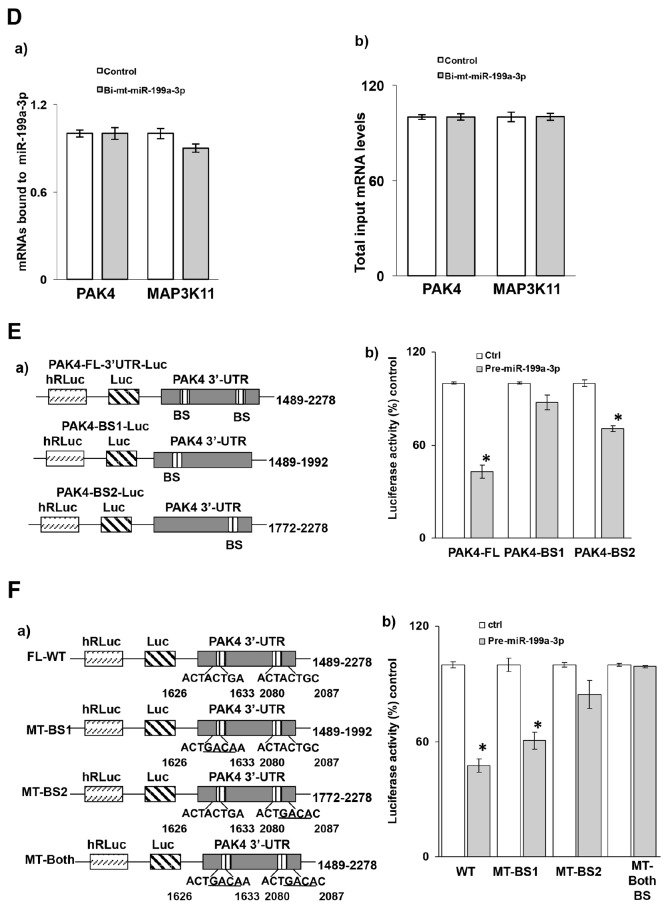
Association of miR-199a-3p with PAK4 mRNA **(A)** Schematic diagram of PAK4 mRNA. (BS) indicates predicted binding sites for miR-199a-3p. **(B)** Levels of (B, a) miR-199a-3p and (b) U6 RNA following transfection of biotinylated-miR-199a-3p (5′ACAGUAGUCUGCACAUUGGUUA 3′Bi, 50 nM) or control biotin-labelled scrambled miR for 48 hrs in the TE7 cells, as measured by q-PCR analysis. Mean ± S.D. of three independent experiments performed in triplicate is shown, and statistical significance is indicated by ^*^ (p<0.001). **(C)** MiR-199a-3p binds to PAK4 mRNA. (C, a) Levels of PAK4 and MAP3K11 mRNAs in the material pulled down by biotinylated-miR-199a-3p and control miR. (C, b) Respective total input mRNA measured by q-PCR. The miR enrichment was calculated as follows: miR-199a-3p pull-down/control-miR pull-down (A), miR-199a-3p input/control-miR input (B), Fold binding = A/B. Representative bar diagram from three separate set of experiments. Each set of experiments was performed in triplicate. Error bars represent mean ±S.D. and ^*^ indicates statistical significance (p < 0.002). (C, c) Copy numbers of PAK4 and Map3K11 mRNAs in the pull-down material mention in (C, a). The levels were measured using dd-PCR. **(D, a)** Levels of PAK4 and MAP3K11 mRNAs in the material pulled down by biotin-labelled mutated miR-199a-3p (5′ CAGACGCCUGCACAUUGGUU A 3′ Bi, 50 nM) and control miR. (D, b) Respective total input mRNA measured by q-PCR. **(E, a)** Schematic representation for PAK4 luciferase reporter constructs containing either the full length 3’UTR (FL-3’UTR) or individual predicted miR-199a-3p binding sites (BS1 or BS2). (E, b) Luciferase activity in the PAK4 reporter constructs following co-transfection with pre-miR-199a-3p (50nM) or control miR in TE7 cells for 36 hours. Luciferase activity in cells transfected with control miR was considered as 100%. Firefly luciferase activity was normalized to Renilla luciferase activity and expressed as the mean of three independent experiments, where all the experiments were carried out in triplicate. Error bars represent mean ± S.D. and ^*^ represents statistically significant (p < 0.05), based on two-tailed Student’s *t* test. **(F, a)** The binding sequence of the miR-199a-3p potential binding sites in (PAK4-full length 3’UTR construct (schematic FL-WT) was mutated either in binding site 1 (schematic, MT-BS1) or in binding site 2 (schematic, MT-BS2) or in both the binding sites (schematic MT-both BS) by substituting 4 bases (underlined). (F, b) Luciferase activity was measured in each construct following co-transfection with pre-miR-199a-3p (50nM) or control miR in TE7 cells for 36 hours. Luciferase activity in cells transfected with control miR was considered as 100%.

The 3’ untranslated region (UTR) of PAK4 mRNA contains two predicted binding sites for miR-199a-3p (Figure 4A). To determine whether miR-199a-3p directly interacts with PAK4 mRNA, following transfection of either biotin-labeled miR-199a-3p or biotin-labelled scrambled miR, cell lysates were exposed to streptavidin-coated beads. RNA was harvested from the pull-down material and amplified with either PAK4 or MAP3K11 probes by q-PCR. MAP3K11 was used as a specificity control in these experiments, as we have previously shown that MAP3K11 binds with miR-199a-5p [13]. As seen in Figure 4C-4a, significant binding was observed between miR-199a-3p and PAK4 mRNA, but not with MAP3K11 mRNA. When analyzed by dd-PCR, there is no difference in the number of MAP3K11 copies in the pull-down material between the control and miR-199a-3p samples, whereas PAK4 copy number is increased by over 8-fold in the miR-199a-3p sample. As an additional control, we also employed biotin-labelled miR-199a-3p in which the predicted PAK4 binding sequence had been mutated. As seen in Figure 4D-4a, there is no difference in binding with either PAK4 or MAP3K11 between control and mutated miR-199a-3p.

To further analyze the interaction between PAK4 mRNA and miR-199a-3p, the full-length PAK4 3’UTR, containing both potential binding sites, as well as two fragments of the 3’UTR each containing one potential binding site, were PCR amplified and separately sub-cloned into a pmirGLO Dual-luciferase miRNA Target expression vector (Figure 4E-4a). Luciferase activity was decreased by approximately 50% following co-transfection of miR-199a-3p with the full-length 3’ UTR construct, by approximately 10% following co-transfection with the construct containing binding site 1, and by approximately 30% following co-transfection with the construct containing binding site 2, compared to control transfection (Figure 4E-4b).

In order to determine the contribution of each potential binding site in mediating the observed effect, site directed mutagenesis was performed to alter four bases in the seed sequence binding region of each predicted miR-199a-3p binding site individually, as well as in both binding sites (Figure 4F-4a). Mutation of binding site 1 had no significant effect on the reduction in luciferase activity seen following co-transfection of the wild type construct with pre-miR-199a-3p. Mutation of binding site 2 significantly abrogated the reduction in luciferase activity, suggesting that this binding site was more critical. Mutation of both binding sites eliminated the decrement in luciferase activity seen with the wild-type construct, suggesting that both binding sites may be required to achieve optimal efficacy (Figure 4F-4b).

### Overexpression of miR-199a-3p decreases TE7 cell proliferation

**Figure 5:**
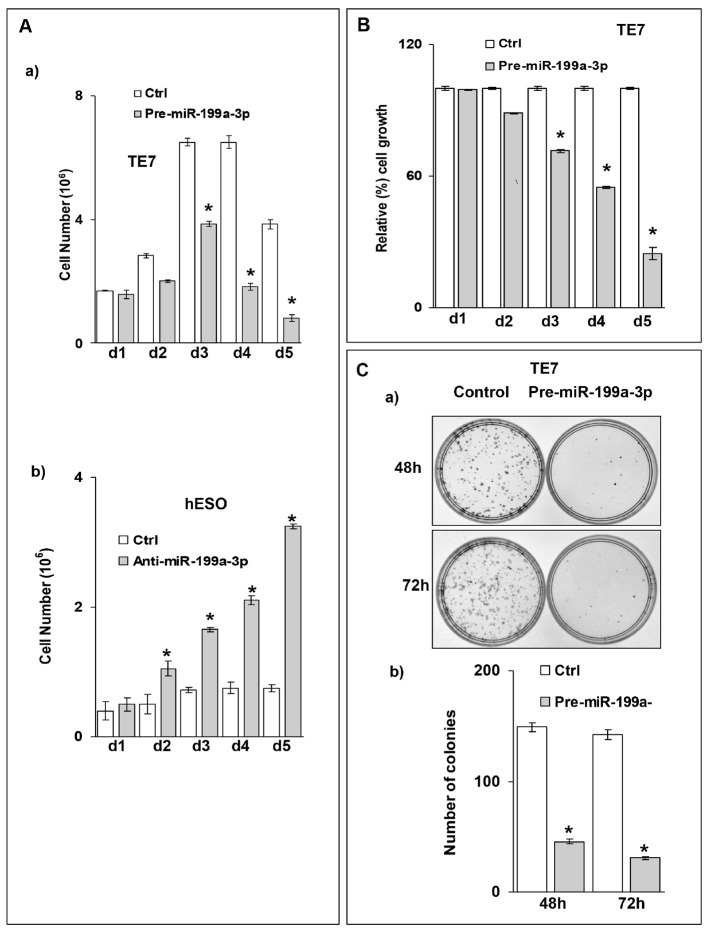
Ectopic expression of miR-199a-3p suppresses cell proliferation in esophageal cancer cells **(A)** Growth assay for TE7 and hESO cells. (A, a) TE7 cells were transfected with 50 nM pre-miR-199a-3p or control miR. (A, b) hESO cells were transfected with 50nM anti-miR-199a-3p or control miR. Post transfection control and transfected cells were counted at indicated time point and live cells were represented on bar diagrams. Error bars represents ± S.D. and statistical significance based on a two-tailed Student’s *t* test is indicated by ^*^ (p < 0.02). **(B)** Cell viability assay for TE7 cells. Pre-miR-199a-3p (50nM) or control miR was transfected into TE7 cells for indicated time. 3-(4, 5-Dimethylthiazol-2-yl)-2, 5-diphenyltetrazolium bromide (MTT) assay was done after each time point. The mean absorbance for cells transfected with control miR was considered as 100%. Values are mean ± SD of three independent experiments in triplicate where ^*^ indicates statistical significance (p< 0.0001). **(C)** Colony formation assay for TE7 cells. (C, a) Pre-MiR-199a-3p (50nM) or control miR was transfected into TE7 cells. Cells were harvested at 48 and 72 hrs, and 2000 cells were reseeded in 60 mm dish for and grown for 14 days. Colonies were stained with crystal violet and counted. (C, b) A bar diagram for number of colonies which grew after two weeks for each time point in control and pre-miR-199a-3p transfected cells shown in (C, a). Statistical significance was calculated based on *t* test and is indicated by ^*^ (p<0.002).

Based on the previously described role of PAK4 in regulating cellular proliferation, we assessed the effect of overexpression of miR-199a-3p on TE7 proliferation. Overexpression of miR-199a-3p results in a significant decrease in TE7 cell proliferation as assessed by cell counts starting at 72 hours following transfection (Figure 5A-5a). Conversely, when miR-199a-3p is silenced in hESO cells, enhanced cellular proliferation is observed starting at 48 hours after transfection (Figure 5A-5b). These results are corroborated by decreased MTT activity in TE7 cells following miR-199a-3p overexpression (Figure 5B). Furthermore, a marked reduction is also observed in colony formation in TE7 cells following either 48 or 72 hours of miR-199a-3p overexpression (Figure 5C).

### MiR-199a-3p reduces TE7 cell proliferation through downregulating PAK4-mediated transcription of Cyclin D1

**Figure 6:**
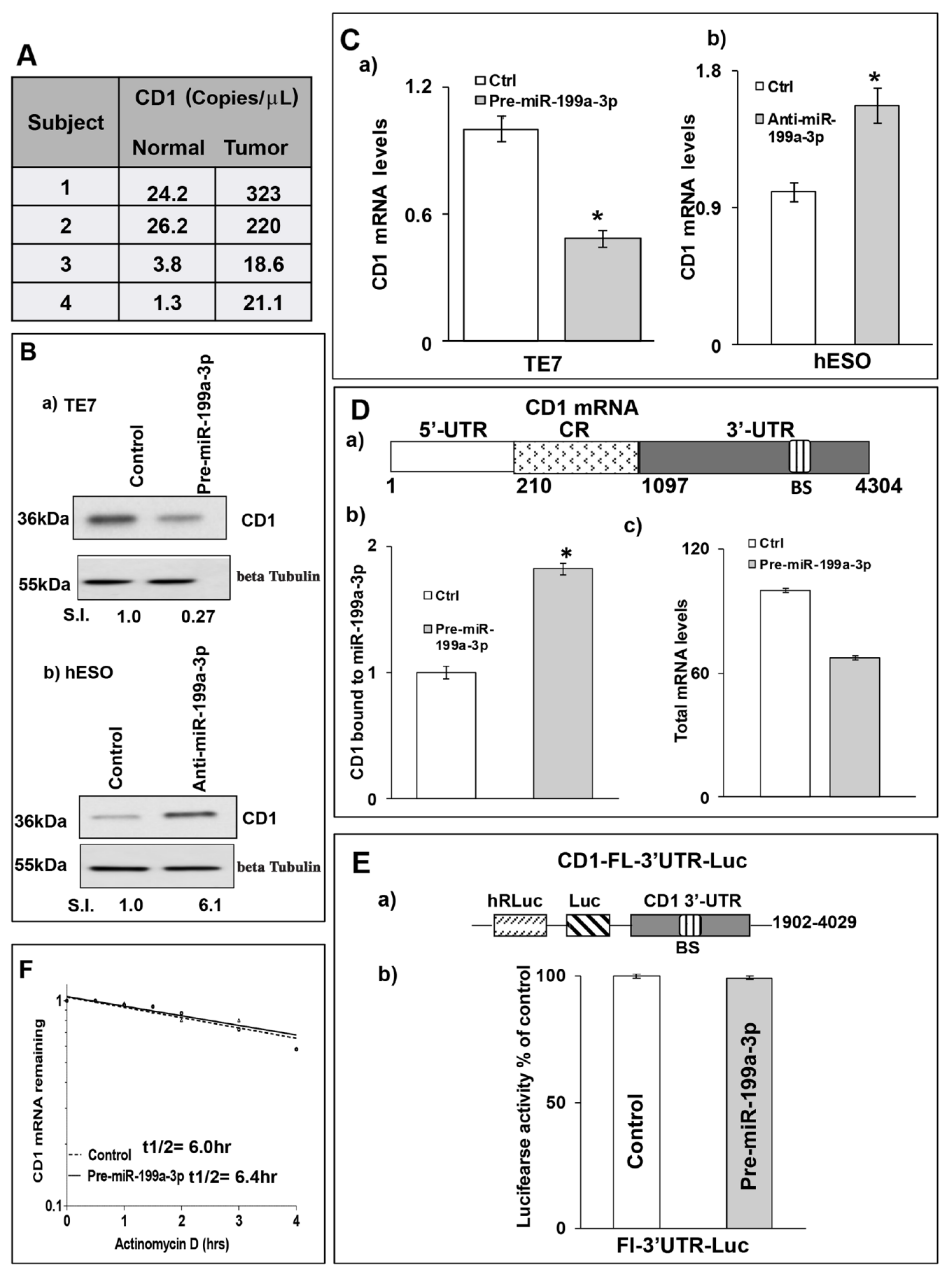
MiR-199a-3p decreases CD1 expression in human esophageal cells **(A)** Copy numbers of CD1 mRNA detected by dd-PCR in human esophageal cancer specimens and matched benign esophageal epithelium. **(B)** Cells were transfected with control or (B, a) 50 nM pre-miR199a-3p (TE7 cells) or (B, b) 50 nM anti-miR-199a-3p (hESO cells) for 48hrs. Post transfection, total cell lysates were subjected to immunoblot analysis for CD1 expression. Beta Tubulin was used to normalize the loading and relative signal intensity was calculated as described above. **(C)** In a similar experiment, levels of CD1 mRNA were measured by q-PCR. Changes in CD1 mRNA levels (C, a) following control miR or pre-miR-199a-3p overexpression in TE7 cells and (C, b) after silencing miR-199a-3p in hESO cells. Values are mean ± SD from three independent sets of experiments performed in triplicate. ^*^ indicates statistical significance (p < 0.002). CD1 mRNA levels were normalized with GAPDH mRNA levels. **(D, a)** Schematic diagram of CD1 mRNA. (BS) indicates a predicted binding site for miR-199a-3p. (D, b) Levels of CD1 mRNA in the material pulled down by biotinylated-miR-199a-3p and control miR measured by q-PCR. (D, c) Levels of CD1 mRNA in total input mRNAs measured by q-PCR in this experiment. The miR enrichment was calculated as described in Figure 4. Error bars represents ± S.D. and statistical significance based on a two-tailed Student’s *t* test is indicated by ^*^ (p < 0.02). **(E, a)** Schematic representation for CD1 luciferase reporter construct containing the full length 3’UTR (FL-3’UTR) with predicted miR-199a-3p binding site (BS). (E, b) Luciferase activity in the CD1 reporter construct following co-transfection with pre-miR-199a-3p (50nM) or control miR in TE7 cells for 36 hours. Measure of luciferase activity was calculated as mentioned earlier in Figure 4. **(F)** Stability of CD1 mRNA in TE7 cells following transfection with either pre-miR-199a-3p or control miR. Total RNA was isolated at indicated time points after administration of Actinomycin D (4μM) and the remaining levels of CD1 mRNA were measured by q-PCR. Levels were normalized with GAPDH. The half-life was calculated as mentioned above in Figure 3B.

We next examined the levels of Cyclin D1 (CD1), a key regulator of cellular proliferation, to investigate the mechanism by which miR-199a-3p overexpression resulted in decreased TE7 cell proliferation. As seen in Figure 6A, CD1 levels are elevated in all four human esophageal cancer specimens compared to matched benign controls. In addition, levels of CD1 are markedly reduced following forced expression of miR-199a-3p in TE7 cells, while levels of CD1 are increased following miR-199a-3p silencing in hESO cells (Figure 6B). As shown in Figure 6C, overexpression of miR-199a-3p in TE7 cells results in a significant decrease in CD1 mRNA, whereas silencing miR-199a-3p in hESO cells leads to an increase in CD1 mRNA level.

Because the 3’ UTR of CD1 mRNA contains a predicted binding site for miR-199a-3p (Figure 6D-6a), we tested whether there may be a direct, functional interaction between miR-199a-3p and CD1 mRNA. In the biotin pull-down experiment depicted in Figure 6D-6b, binding was observed between miR-199a-3p and CD1 mRNA. To determine whether the observed binding interaction had functional significance, a luciferase reporter construct containing the full-length 3’ UTR of CD1 mRNA was co-transfected with miR-199a-3p. Interestingly, there was no reduction in luciferase activity compared to co-transfection with control miR (Figure 6E), suggesting that the observed binding interaction was non-functional. In addition, no change in CD1 mRNA stability was seen in TE7 cells following miR-199a-3p overexpression (Figure 6F).

**Figure 7:**
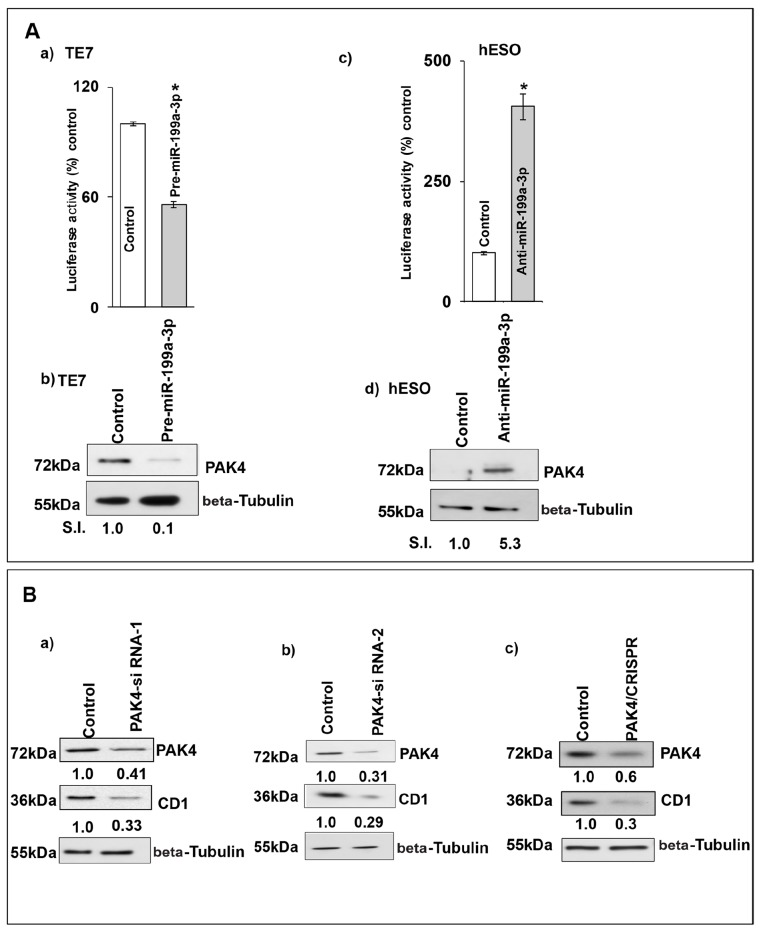

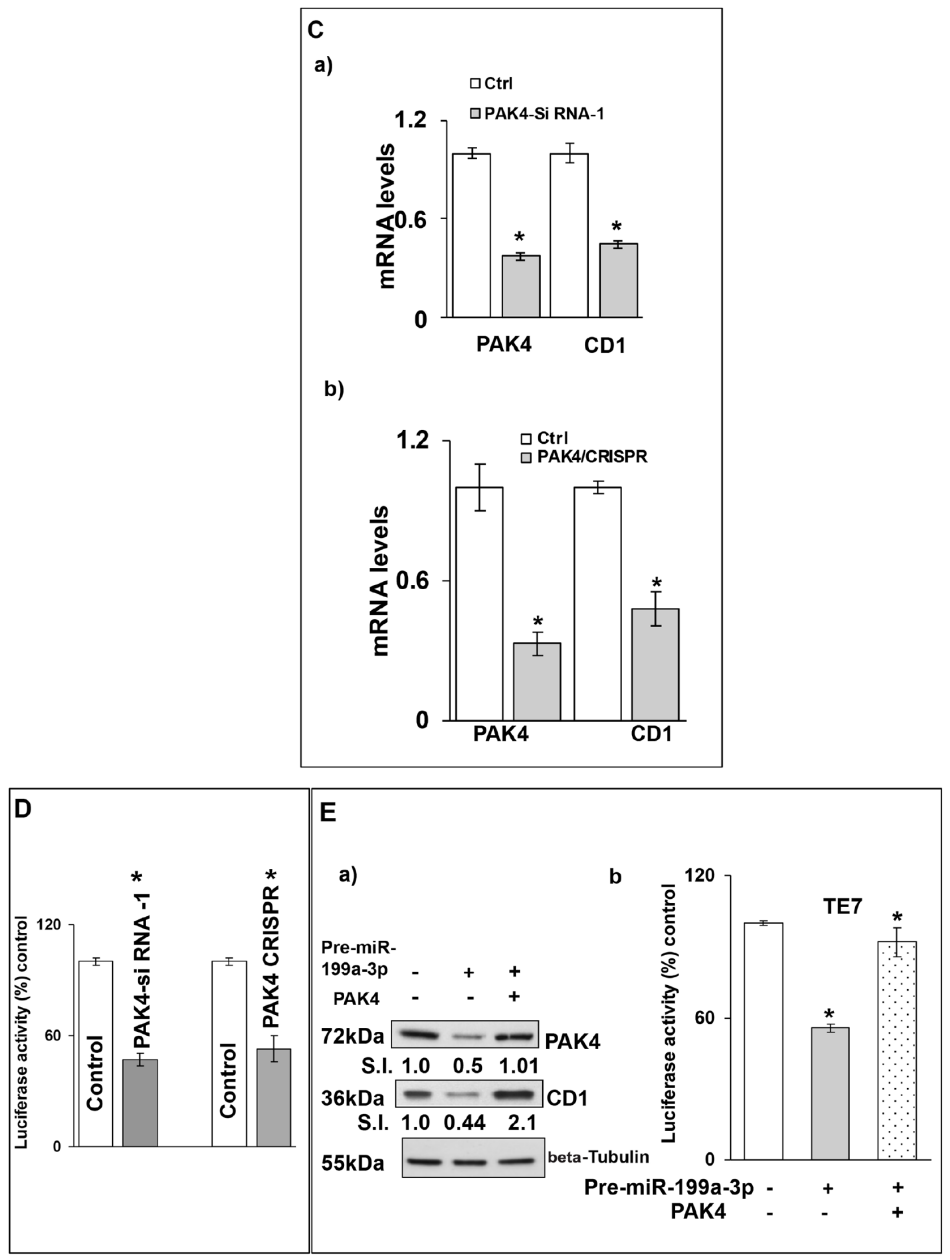
Silencing PAK4 inhibits CD1 transcription **(A)** Changes in CD1 promoter activity after co-transfection of CD1-promoter (100 ng, 1748 CD1 promoter PGL3 basic, # 32726) luciferase reporter construct, pRL-TkRenilla (10 ng, Promega) with either control miR, (A, a) pre-miR-199a-3p (50nM) or (A, c) anti-miR-199a-3p (50nM). Firefly luciferase activity was normalized to Renilla luciferase activity and expressed as the mean of three independent experiments, where all the experiments were carried out in triplicate. Luciferase activity in cells transfected with control miR is considered 100%. Error bars represent mean ± S.D. and ^*^ represents statistical significance (p < 0.0001). (A, b and d) The corresponding PAK4 protein levels in whole cell lysates derived from the cells used for the CD1 promoter activity assay shown in (A, a and c) respectively. **(B)** TE7 cells were transfected with control scrambled siRNA or (B, a) PAK4-siRNA-1, (B, b) PAK4-siRNA-2, (B, c) PAK4 CRISPR/Cas9 KO plasmid. The total cell extract was made after 48 hrs and subjected to Western blot analysis for PAK4 and CD1. Beta Tubulin was used as a loading control. Signal intensity was determined as described in Figure 1. **(C)** Changes in levels of PAK4 and CD1 mRNAs post-transfection of control, scrambled siRNA or (C, a) PAK4 siRNA-1, (C, b) PAK4 CRISPR/Cas9KO plasmid. Total RNA was isolated and levels of PAK4, CD1 and GAPDH were determined by q-PCR. mRNA levels for cells transfected with control scrambled siRNA were set as 100%. Statistical significance calculated by *t* test and represented by ^*^ p<0.001. **(D)** Changes in CD1 promoter activity in PAK4 silenced TE7 cells using PAK4 siRNA-1 or PAK4 CRISPR/Cas9KO plasmid. Promoter activity was measured as described in (A) Luciferase activity for cells transfected with control, scrambled siRNA was set as 100%. ^*^ indicates statistical significance p<0.0001. **(E)** PAK4 rescues CD1 levels. (E, a) Following overexpression of pre-miR-199a-3p (50nM) in TE7 cells (middle & last lane) cells were transfected with 3μg PAK4 plasmid (last lane). Levels of PAK4 (top panel) and CD1 (middle panel) were measured by Western blot. Levels of target proteins were normalized by Beta Tubulin (bottom panel). SI indicates relative signal intensity of PAK4 and CD1. Relative signal intensity is determined as explained in Figure 1B, d. (E, b) Changes in CD1 promoter activity in the TE7 cells after co-transfection of CD1 promoter with PAK4 plasmid and/or pre-miR-199a-3p as described in (E, a). Mean ± S.D. of three independent experiments performed in triplicate is shown. Statistical significance is indicated by^*^ (p<0.0001).

The lack of change in mRNA stability despite a reduction in CD1 mRNA levels, led us to postulate that miR-199a-3p may regulate CD1 expression by reducing its transcription. To investigate this possibility, we measured CD1 promoter activity following miR-199a-3p overexpression. Co-transfection of pre-miR-199a-3p with a luciferase reporter construct containing the CD1 promoter resulted in a significant reduction in luciferase activity compared to transfection with control miR (Figure 7A-7a). Conversely, silencing miR-199a-3p in hESO cells resulted in a significant increase in CD1 promoter activity compared to transfection with control miR (Figure 7A-7c). These changes in CD1 promoter activity were correlated with changes in PAK4 protein expression (Figure 7A-7b, 7d).

To determine whether this effect on CD1 transcription was mediated by PAK4, we silenced PAK4 with two distinct siRNAs as well as with a CRISPR-PAK4 knock-out construct and saw decreased CD1 protein and mRNA expression with all approaches (Figure 7B and 7C). Importantly, significantly decreased CD1 promoter activity was also seen after PAK4 silencing (Figure 7D). Finally, in the rescue experiment depicted in Figure 7E, when PAK4 is overexpressed following transfection of pre-miR-199a-3p, CD1 expression is restored. This restoration of CD1 protein expression is associated with restored CD1 promoter activity.

## DISCUSSION

Our findings indicate that miR-199a-3p is markedly downregulated in human esophageal cancer specimens and cell lines compared to esophageal epithelial cells. We demonstrate that miR-199a-3p functions as a tumor suppressor in esophageal cancer cells by regulating PAK4 expression through a direct interaction with its mRNA. Forced expression of miR-199a-3p leads to a decrease in PAK4 mRNA and protein levels through decreased mRNA stability. Although not investigated in this study, it may also be possible that miR-199a-3p affects PAK4 translation. Finally, this downregulation of PAK4 results in decreased transcription of CD1 which contributes to impaired cellular proliferation. Although overexpression of miR-199a-3p and downregulation of PAK4 may affect proliferation through other mechanisms not evaluated in this study, our findings support an important role for CD1 in mediating the observed decreased proliferation.

In previous histopathologic studies, CD1 has been found to be overexpressed in 40-70% of esophageal cancer cases analyzed [14–15]. Furthermore, the degree of CD1 overexpression has been shown to be associated with adverse pathologic factors such as poor differentiation and advanced stage [16–18]. To date, investigations into the mechanisms regulating CD1 expression in esophageal cancer cells have focused mainly on its transcriptional control, which is regulated by multiple pathways [19]. Not surprisingly, data exists on the activation of several pathways resulting in increased CD1 expression in esophageal cancers cells, including the AKT, Notch, and NF-κB pathways [20–22]. PAK4 has previously been shown to affect the transcription of CD1 through the inhibition of NFκB activity by preventing its nuclear translocation [10]. Other investigators have linked PAK4 to CD1 through the beta-catenin pathway [23].

In addition to providing an initial description of the role of miR-199a-3p in esophageal cancer cells, these data also describe an important role for PAK4 in esophageal cancer. PAK4 is highly expressed in embryonic tissues, but not expressed in normal adult tissues and has proven to be tumorigenic in mice [24–25]. PAK4 has been previously shown to be overexpressed in human esophageal squamous cell cancers compared to paired normal esophageal epithelium [25]. In this study, samples from four patients were examined by Western Blot analysis of whole cell lysates, with PAK4 overexpression demonstrated in three of the samples. In our analysis of human tumor samples, we saw elevation of PAK4 in all four patient tumor samples compared to matched benign esophageal epithelial controls as assessed by copy number. Also, all four patients also demonstrated reduced levels of miR-199a-3p in the tumor samples. Notably, the differences in PAK4 mRNA levels are more pronounced than the differences in miR-199a-3p levels, suggesting that smaller changes in miR levels may result in more marked changes in target mRNA expression.

Studies such as these highlight the potential of miR analysis to identify new targets with potential therapeutic value in specific malignancies. This is especially true for PAK4, as small molecule inhibitors of this kinase have been identified. The PAK4-inhibitor KPT-9274 has been shown to have efficacy in renal cell cancer lines [23]. This is agent is currently undergoing clinical Phase 1 testing in solid tumors and lymphomas. A second PAK4-inhibitor, termed PF-3758309 has also demonstrated anti-neoplastic efficacy through decreasing cellular proliferation in breast cancer cells [26].

Finally, these data support an important role for the miR-214-3p/miR-199a-5p/3p cluster in esophageal carcinogenesis. MiR-199a2, which is located on chromosome 1, encodes pri-miR-199a, as well as the precursor sequences for miR-214 [27]. This cluster has been shown to be dysregulated in hepatocellular cancer and pancreatic cancer [28–29]. Our esophageal cancer cell line array analysis has shown that miR-214-3p, miR-199a-5p, and miR-199a-3p are 3 of the most downregulated miRs in the cancer cells [4]. We have previously shown that miR-214-3p regulates expression of the RNA-binding protein CUG-BP1 as well as the anti-apoptotic protein survivin in these cells [4]. MiR-199a-5p regulates expression of MAP3K11, which also modulates transcription of CD1 in these cells [13]. Going forward, determining both the frequency with which this cluster is downregulated in human specimens as well the mechanism by which this occurs will yield important information regarding the development of esophageal cancer. It is plausible to envision that deactivation of this cluster may be an early step in esophageal carcinogenesis and could potentially identify patients with Barrett’s dysplasia who are at high risk for progression to cancer.

## MATERIALS AND METHODS

### Cell culture and reagents

The human esophageal squamous cancer cell lines TE7, TE10 and human esophageal epithelial cell line hESO were obtained and maintained as explained previously [4]. The human esophageal adenocarcinoma cancer cell line FLO1 was purchased from European Collection of Authenticated cell culture (England, UK). Cells were cultured in RPMI media (Mediatech Inc, Manassas, VA, USA) supplemented with 10% heat-inactivated FBS and maintained in a 37°C humidified incubator with 5% CO_2_.

### Transfection

All transfections were done for 48 hrs as described earlier [4]. Briefly, 0.5-0.75 × 10^6^cells were seeded in 60 mm plates a day prior to transfection. For miR transfections, pre-miR-199a-3p [(50 nM), miR base id-hsa-miR-199a-3p and assay id PM-11779], anti-miR-199a-3p [(50 nM), miR base id-hsa-miR-199a-3p and assay id AM-11779], or control miR (Ambion, Austin, TX, USA) were diluted in 500μl Opti-MEM (Invitrogen, Carlsbad, CA, USA). Diluted miRNA was mixed with 5 μl Lipofectamine RNAiMAX (Invitrogen, Carlsbad, CA, USA) and incubated at room temperature (RT) for 15 minutes. The complex was added to the cells in a final volume of 5 ml of fresh medium. In overexpression experiments, 3μg PAK4 plasmid (OriGene, Rockville, MD, USA) was utilized. For RNA silencing, 80 pmol of either PAK4-siRNA-1[Santa Cruz Biotechnologies (catalog no sc39060)] or PAK4-siRNA-2 [Dharmacon Inc (catalog no L003615-00)] were used. Each of these siRNAs contain pools of 3-5 target-specific PAK4 siRNAs. In additional silencing experiments, 0.25 μg PAK4CRISPR/CAS9 KO plasmid (Santa Cruz, Dallas, TX, USA) was used. Prior to transfection, 0.25 × 10^6^ cells were seeded in duplicate in 24 well plates. Next day, transfection was performed with the various reagents described above with either 5 μl Lipofectamine 2000 (Invitrogen, Carlsbad, CA, USA) for the DNA-based reagents or 5 μl Lipofectamine RNAiMAX for the siRNAs in 500μl Opti-MEM. Medium was replaced 8 hrs post transfection and cells were grown for additional 2 days. After that cells were pelletted for lysis. For PAK4CRISPR/CAS9 KO plasmid, pellets from both the wells were pooled together for lysis. Protein estimation was done using BCA kit and immunoblots were performed as mentioned below.

### Reverse transcription (RT) and quantitative real-time PCR (q-PCR) analyses

All RT and q-PCR experiments were performed as described earlier [4]. Q-PCR was performed in triplicate with specific (CD1, PAK4, MAP3K11, miR-199a-3p, U6 and GAPDH) TaqMan primers and probes (Applied Biosystems, Foster City, CA, USA). The levels of GAPDH were used to normalize levels of CD1 and PAK4 in q-PCR samples. For miR experiments, normalization was accomplished using small nuclear RNA U6.

### Esophageal cancer specimens

Biopsies of esophageal tumor as well as adjacent non-malignant epithelium with no gross evidence of tumor or Barrett’s esophagus were obtained at the time of esophagectomy from 4 patients enrolled in an IRB-approved protocol. None of the patients were treated with chemoradiotherapy prior to surgery. Samples were snap-frozen in liquid nitrogen prior to processing. The tissues were ground to a fine powder using mortar and pestle without allowing them to thaw. Tissue powder was homogenized using QIAshredder (Qiagen, Valencia, CA, USA) in lysis buffer and total RNA was extracted as mentioned above.

### Droplet digital PCR (dd-PCR)

Droplet Digital PCR (ddPCR^™^) was performed using the QX200^™^ ddPCR^™^ system (Bio-Rad, Hercules, California). All reagents, disposables, and equipment are from Bio-Rad except the probe. The droplets were generated for each sample PCR reaction mixture using Droplet Generation Oil. Then C1000^™^ thermal cycler was used with cycling conditions 95°C for 10 minutes followed by 40 cycles of 94°C for 30 seconds and 60°C for one minute, followed by 98°C for 10 minutes. Plate was then transferred to the QX200^™^ Droplet Reader and the data were analyzed using QuantaSoft^™^ Software version 1.7.

### Immunoblotting

Whole cell lysates were resolved on 10% SDS-PAGE gels (Bio-Rad Laboratories, Hercules, CA, USA) and Immunoblots were performed as reported earlier [4]. Anti-human PAK4 antibody was purchased from Cell Signaling (Danvers, MA, USA), anti-CD1 was purchased from Millipore (Billerica, MA, USA), anti-GAPDH, anti-tubulin, and horseradish peroxidase-conjugated anti-mouse or anti-rabbit antibodies were purchased from Santa Cruz Biotechnology (Dallas, TX). Signal intensity was quantified using Image Lab quantification software (Bio-Rad, Hercules, CA, USA).

### Bioinformatics

Four software programs, RegRNA 2.0 (regrna.mbc.nctu.edu.tw), Weizmann Institute of Science (http://genie.weizmann.ac.il/index.html), TargetScan Human (http://www.targetscan.org) and miRDB (http://mirdb.org/miRDB) were used to predict the potential target genes of miR-199a-3p.

### mRNA stability

mRNA stability assays were performed as previously reported [4, 30].

### Biotin-labeled pull-down assays

Biotinylated miR-199a-3p ((5′ ACAGUAGUCUGCACAUUGGUUA 3′Bi), pull-down assays with target mRNAs were performed as described earlier [4, 31]. For biotin labeled mutated miR-199a-3p, 7 bases of miR-199a-3p (5′CAGACGCCUGCACAUUGGUUA 3′Bi) complementary to seed region of PAK4-3’UTR binding sequences were mutated (underlined). Biotinylated transcripts were synthesized from Dharmacon, Lafayette, CO, USA.

### Luciferase reporter assay

Luciferase reporter constructs were prepared as previously described [4]. For CD1 (NM_053056.2) and PAK4 (Variant 5, NM_001014834.2) full-length 3’ UTR luciferase reporter constructs were generated. PCR amplified individual insert fragments were sub-cloned into a SacI and Xba1 or SacI and DraI (New England Bio Labs, Ipswich, MA, USA) digested pmirGLO Dual-Luciferase miRNA target expression vector (Promega, Madison, WI, USA). The PAK4 constructs containing mutations at the sequence of binding region of potential binding sites were generated using a site directed mutagenesis kit (Agilent Technologies, Santa Clara, CA, USA). All primer sequences used to create these constructs are listed in Table 1. The orientation and the sequence of the constructs were confirmed by restriction enzyme digestion and DNA sequencing.

**Table 1:** Primer sequences used to generate luciferase reporter constructs for miR-199a-3p binding studies

Name	Sequence	Region	Enzyme
pmirGLO-PAK4-FL-UTR-Fwd	**TTTAAA**CCCTTCCCCTCAACCAAAGA	1489-2278	DraI
pmirGLO-PAK4-FL-UTR-Rev	**GAGCTC** AAACTAACTCGAGGCAGGGG	1489-2278	SacI
pmirGLO-PAK4-BS1-Fwd	**TTTAAA**CCCTTCCCCTCAACCAAAGA	1489-1992	DraI
pmirGLO-PAK4-BS1-Rev	**GAGCTC**GGCCACTCTTCGGACATTCA	1489-1992	SacI
pmirGLO-PAK4-BS2-Fwd	**TTTAAA**CACTGGAAGTCTGCAGTGGG	1772-2278	DraI
pmirGLO-PAK4-BS2-Rev	**GAGCTC**AAACTAACTCGAGGCAGGGG	1772-2278	SacI
pmirGLO-PAK4-UTR-BS1-Mt-Fwd	GGGGTAGATGAGACCCTACTGACAAAC TCCAGTTTTGATCTCGTG	1489-2278	N/A
pmirGLO-PAK4-UTR-BS1-Mt-Rev	CACGAGATCAAAACTGGAGTTTGTCAG TAGGGTCTCATCTACCCC	1489-2278	N/A
pmirGLO-PAK4-UTR-BS2-Mt-Fwd	CCCCTGCAGCAAATGACTGACACACCT GGACAGCCTCCTC	1489-2278	N/A
pmirGLO-PAK4-UTR-BS2-Mt-Rev	GAGGAGGCTGTCCAGGTGTGTCAGTCA TTTGCTGCAGGGG	1489-2278	N/A
pmirGLO-CD1-FL-UTR-Fwd	**GAGCTC**GTCCCACTCCTACGATACGC	1902-4092	SacI
pmirGLO-CD1-UTR-Rev	**TCTAGA** CCTTTGGCCTCTCGATACAC	1902-4092	XbaI

1748 human cyclin D1 promoter pGL3Basic was a gift from Frank McCormick (Addgeneplasmid # 32726) and promoter activity was measured as described previously [13, 32].

### Cell proliferation studies

Cell proliferation assay was done as described previously [13]. Briefly, cells were transfected for 1-5 days with pre-miR-199a-3p then incubated with 3-(4-5-dimehtylthiazol-2-yl)-2,5-diphenyltetrazolium bromide (MTT, Roche, Mannheim, Germany) for ∼4 h at 37°C. Medium was removed and dimethyl sulfoxide was added. Optical density was measured at 550nM. For cell growth study, cells were seeded a day before transfection. Cells were counted on days through 5 post-transfection.

### Colony formation assay

TE7 cells were transfected with pre-miR-199a-3p as mentioned above. Following transfection, 2000 control and transfected cells were seeded in 60 mm dishes to grow for 14 days. Colonies were then stained with crystal violet and counted.

### Statistical analysis

Results are expressed as the means ± S.D from three independent experiments with minimum three replicates for each set of experiment. Data derived from multiple determinations were subjected to two-tailed Student’s *t* test and p values < 0.05 were considered significant.
